# Near Infrared Fluorophore-Tagged Chloroquine in *Plasmodium falciparum* Diagnostic Imaging

**DOI:** 10.3390/molecules23102635

**Published:** 2018-10-14

**Authors:** Li Yan Chan, Joshua Ding Wei Teo, Kevin Shyong-Wei Tan, Keitaro Sou, Wei Lek Kwan, Chi-Lik Ken Lee

**Affiliations:** 1 Department of Technology, Innovation and Enterprise (TIE), Singapore Polytechnic, 500 Dover Road, Singapore 139651, Singapore; CHAN_Li_Yan@sp.edu.sg (L.Y.C.); Joshua_TEO@sp.edu.sg (J.D.W.T.); 2 Laboratory of Molecular and Cellular Parasitology, Department of Microbiology and Immunology, National University of Singapore, 5 Science Drive 2 Block MD4, Level 3, Singapore 117545, Singapore; mictank@nus.edu.sg; 3 Research Institute for Science and Engineering, Waseda University, 3-4-1 Ohkubo, Shinjuku-ku, Tokyo 169-8555, Japan; soukei@aoni.waseda.jp; 4 Engineering Product Development, Singapore University of Technology and Design, 8 Somapah Road, Singapore 487372, Singapore; kwanwl@sutd.edu.sg

**Keywords:** squaraine dye, near infrared, fluorescence, chloroquine, malaria, *Plasmodium falciparium*

## Abstract

Chloroquine was among the first of several effective drug treatments against malaria until the onset of chloroquine resistance. In light of diminished clinical efficacy of chloroquine as an antimalarial therapeutic, there is potential in efforts to adapt chloroquine for other clinical applications, such as in combination therapies and in diagnostics. In this context, we designed and synthesized a novel asymmetrical squaraine dye coupled with chloroquine (SQR1-CQ). In this study, SQR1-CQ was used to label live *Plasmodium falciparum* (*P. falciparum*) parasite cultures of varying sensitivities towards chloroquine. SQR1-CQ positively stained ring, mature trophozoite and schizont stages of both chloroquine–sensitive and chloroquine–resistant *P. falciparum* strains. In addition, SQR1-CQ exhibited significantly higher fluorescence, when compared to the commercial chloroquine-BODIPY (borondipyrromethene) conjugate CQ-BODIPY. We also achieved successful SQR1-CQ labelling of *P. falciparum* directly on thin blood smear preparations. Drug efficacy experiments measuring half-maximal inhibitory concentration (IC50) showed lower concentration of effective inhibition against resistant strain K1 by SQR1-CQ compared to conventional chloroquine. Taken together, the versatile and highly fluorescent labelling capability of SQR1-CQ and promising preliminary IC50 findings makes it a great candidate for further development as diagnostic tool with drug efficacy against chloroquine-resistant *P. falciparum*.

## 1. Introduction

Malaria is a deadly disease caused by the protozoan parasite *Plasmodium falciparum* that affects resource-limited regions such as Africa and South-east Asia [1]. Chloroquine (CQ) was the drug of choice against malaria due to its clinical effectiveness and low-cost. However, mutations within the parasite quickly gave rise to CQ resistance and CQ was taken off the arsenal of frontline drugs against malaria [2]. In recent years, several groups were focusing on resolving the problem of CQ resistance in malaria, such as by seeking ways towards new uses of CQ in practical applications against malaria [2,3,4,5]. Some of these novel approaches involve modification of CQ and related compounds towards fluorescence-based diagnostic applications [3,6,7]. Of several different classes of fluorescent compounds assessed for staining of malaria parasites, organic near infrared (NIR) fluorescent dyes present as promising candidates.

NIR fluorescent dyes have been studied with increasing interest for use in imaging and diagnostics due to their flexibility to bind further with various kinds of specific molecules, such as chemical small molecules, amino acids, proteins, nucleotides, DNA primers, double-stranded DNA and antibodies [8,9,10,11,12,13]. However, there are only a few readily available NIR dyes, such as, borondipyrromethenes (BODIPYs), cyanines, squaraines, benzo[c]heterocycles, xanthenes, phthalocyanines and porphyrins [14,15]. Squaraine dyes are good candidates for photodynamic therapy, bioimaging and biochemical labeling, as its NIR absorption and emission range fall outside the self-absorption and self-fluorescence region of biological media [16,17,18,19]. Compared to the classical symmetrical squaraines, asymmetrical squaraines are considerably less investigated, due in part to the more complex preparation of asymmetric derivatives. In our previous study, we have discussed how dibutylaniline is an important moiety of asymmetrical squaraine dyes that contributed to a significant larger Stoke shift [20]. Large Stoke shift of the asymmetrical squaraine dyes is desirable in their fluorescence labeling applications as it reduces self-quenching effects and interference from excitation source.

In this study, building on this important finding, we herein designed and synthesized 2 squaraine NIR dyes SQR1 and SQR2 and explored their potential. The dyes were then functionalized via conjugation with CQ for the CQ-tagged derivative compounds SQR1-CQ and SQR2-CQ, which could be developed as potential malaria drug or diagnostic probes.

## 2. Results and Discussion

### 2.1. Synthesis of Squaraine NIR Dye Conjugated with Chloroquine

**Scheme 1:**
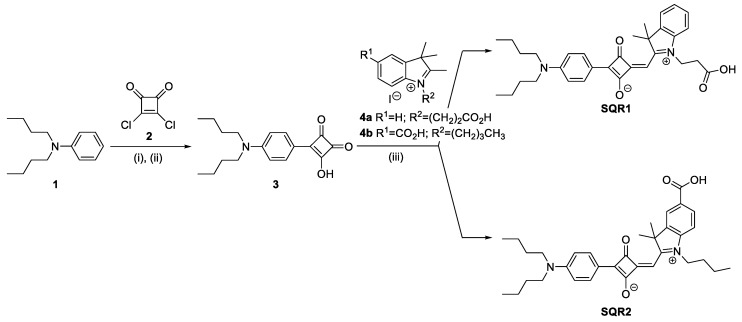
Synthetic route of squaraine near infrared (NIR) dye. Reagents and conditions: (**i**) toluene, reflux, 6 h; (**ii**) 5N HCl, AcOH/H_2_O (1:1), reflux, 2 h, 67% yield over 2 steps; (**iii**) *n*-BuOH/toluene (4:1); reflux; 3 h, SQR1: 49% yield, SQR2: 45%.

**Scheme 2:**
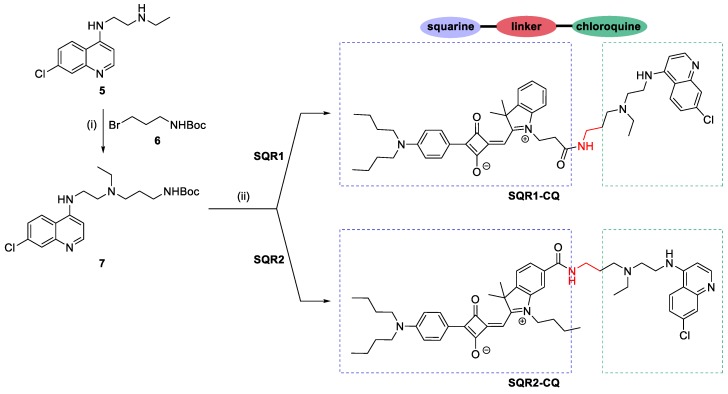
Synthetic route of squaraine-chloroquine dye. Reagents and conditions: (**i**) *N*,*N*-Diisopropylethylamine (DIPEA), *N*,*N*-Dimethylformamide (DMF), 90 °C, 20 h, 85% yield; (**ii**) TFA, 0 °C to rt, 3 h; HATU, DIPEA, CH_2_Cl_2_, rt, 20 h, SQR1-CQ: 45%, SQR2-CQ: 42%.

The synthesis of squaraine NIR dye, SQR1 and SQR2, is outlined in Scheme 1. The design contains three fragments–aniline, squaric core and indolium. The sequence in preparing the semi-squaraine intermediate is crucial in order to successfully form the asymmetrical squaraine dyes. The precursors *N*,*N*-dibutylaniline **1** [21] and squaryl chloride **2** [22] were prepared based on previously reported procedures. The coupling of **1** and **2** under refluxing toluene gave the chloride intermediate, which was then hydrolyzed in a one-pot fashion to obtain squaric acid **3**. The two different indolium salts, **4a** [23] and **4b [24]**, were synthesized using previously published procedures. Reacting **3** and **4** together in a 4:1 mixture of refluxing *n*-butanol/toluene yielded the respective squaraine dyes. However, the carboxylic group of the squaraine dyes reacted with the *n*-butanol solvent to form the butyl ester derivatives instead. This could be easily resolved by a simple HCl acid hydrolysis of the butyl esters to revert back to SQR1 and SQR2, respectively. It is interesting to note that by using a basic hydrolysis of Na^*t*^OBu in THF instead of acid hydrolysis, the –(CH_2_)_2_CO_2_Bu attached to the indolium was cleaved. This dye candidate, when embedded within folic acid functionalized liposome membrane, could be used as ovarian cancer cells bioimaging [25]. The presence of the carboxylic group is an important design for further functionalization of the squaraine NIR dyes. In this report, the squaraine dyes were coupled with chloroquine to serve as a malaria diagnostic probe, which is depicted in Scheme 2. **5** was synthesized from known method [26] and reacted readily with Boc protected **6** [27] to give modified CQ **7**. By combining either SQR1 or SQR2 to CQ **7**, SQR1-CQ or SQR2-CQ were formed via amide bond coupling using HATU.

### 2.2. Labelling Applications of SQR1-CQ on P. falciparum

Given the superior fluorescence performance of SQR1-CQ (Figure S1), SQR1-CQ was the focus for further characterization in this study. To date, other fluorophore-tagged CQ combinations have been synthesized and investigated in cellular labelling applications [1,2]. However, there have been no reports assessing novel squaraine dyes for use in fluorescence staining of *P. falciparum*. We thus set out to investigate cellular labelling capability of SQR1-CQ in several strains of *P. falciparum* and report that at a low concentration of 1 µM, SQR1-CQ exhibited consistent labelling capability of multiple *P. falciparum* erythrocytic cell stages, with similar localization of SQR1 in each of these stages. Both CQ-sensitive (3D7) and CQ-resistant (7G8 and K1) *P. falciparum* strains were used. Although other fluorescent compounds have been synthesized and assessed for labelling of *P. falciparum* towards diagnostics applications, the groups behind these reports had either only used CQ-sensitive *P. falciparum* strains [28] or *Plasmodium* species that is not known to infect humans [29]. Recently, Loh et al. had quantitatively reported uptake of a CQ-BODIPY conjugate by 3D7, 7G8 and K1 strains [1]. We adapted this group of *P. falciparum* strains for this study and presented visual labelling profiles of SQR1-CQ for all three strains.

**Figure 1:**
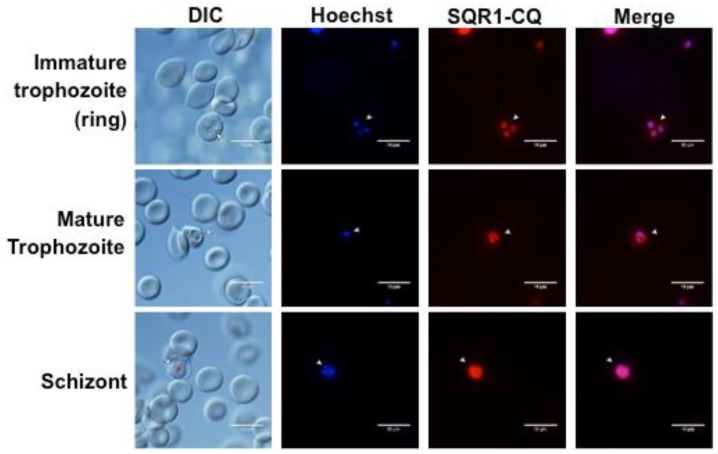
Squaraine dye coupled with chloroquine (SQR1-CQ) labelling of CQ-sensitive *P. falciparum*. Mixed in vitro cultures of 3D7 *P. falciparum* were co-labelled with nuclear stain Hoechst 33342 and SQR1-CQ, before analyzing via confocal microscopy. We observe multiple stages of 3D7 (arrowheads) exhibiting uptake and labelling with both dyes. Hoechst 33342 indicated localization of nuclei in *P. falciparum* stages while SQR1-CQ was shown to specifically sequester within cytoplasmic space of 3D7 and not in RBCs. Surrounding unparasitized RBCs we not observed to retain SQR1-CQ or Hoechst 33342. Bar = 10 µm.

We first incubated SQR1-CQ to CQ-sensitive *P. falciparum* 3D7 parasite cultures at different stages of the parasite erythrocytic cycle, from immature ring-form trophozoites to mature schizonts. SQR1-CQ was capable in labelling 3D7 parasites strongly at each of the three stages (Figure 1). Co-staining with nuclear stain Hoechst 33342 showed that SQR1-CQ did not co-localize with Hoechst 33342 in the nucleus and occupied the cytoplasmic space of the parasite cells.

**Figure 2:**
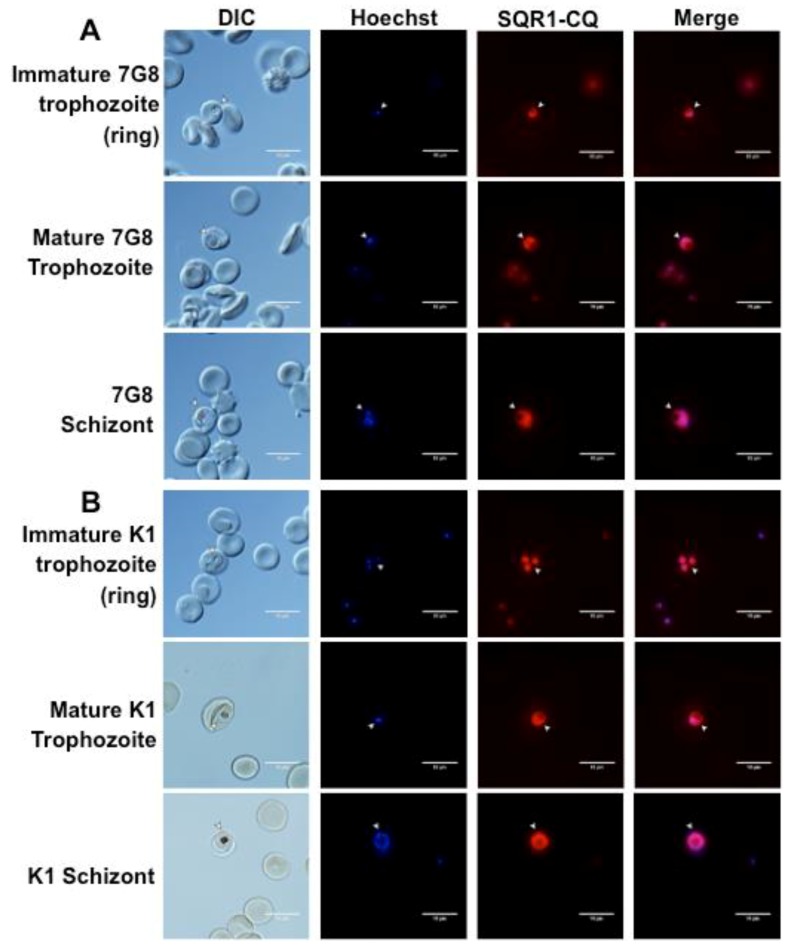
SQR1-CQ labelling of CQ-resistant *P. falciparum*. Mixed in vitro cultures of (**A**) 7G8 and (**B**) K1 *P. falciparum* were co-labelled with nuclear stain Hoechst 33342 and SQR1-CQ, before analyzing via confocal microscopy. Similar to 3D7, multiple stages of both intermediate and high resistance strains (arrowheads) exhibiting uptake and labelling with both dyes. Localization of SQR1-CQ in 7G8 and K1 does not differ from that observed in 3D7. Bar = 10 µm.

We then proceeded to perform SQR1-CQ labelling on CQ-resistant *P. falciparum* strains, 7G8 (intermediate resistance) and K1 (high resistance) using a similar staining protocol as with 3D7. The localization of SQR1-CQ in 7G8 and K1 parasite cells, from immature ring-form trophozoites to mature schizonts, was similar to that observed in 3D7 (Figure 2). Accumulation of SQR1-CQ in the cytoplasmic space, outside of the food vacuole, was observed consistently across all three *P. falciparum* strains. These demonstrated the versatility of SQR1-CQ in labelling various strains and cell morphologies of *P. falciparum*, notably the successful labelling of immature ring-form trophozoites, which is considered the most commonly observed stage [30].

Interestingly, SQR1-CQ was not observed to accumulate in the food vacuole of the erythrocytic stages of *P. falciparum* as seen in the analysis of fluorescence imaging with differential interference contrast (DIC) micrographs of Figure 1 and 2. This is in contrast to the mechanism of CQ as an antimalarial, in which its uptake and accumulation into the food vacuole inhibits digestion of hemoglobin for nutrients [3]. Chloroquine, as a weak base, passively diffuses into *P. falciparum* erythrocytic cell stages in its neutral state. It gets protonated after entering an acidic compartment such as the food vacuole and is increasingly sequestered there due to the protonated form being membrane impermeable [3,4,5]. We hypothesized that unlike untagged CQ, SQR1-CQ does not accumulate in the food vacuole due to the presence of the squaraine moiety. It is reported that the resting cytoplasmic pH of *P. falciparum* trophozoites is at approximately 7.3 [6,7]. At this regulated neutral pH, we hypothesized that SQR1-CQ, a stronger base than untagged CQ, exists as a positive charged species in the cytosol. In this state, it is likely that SQR1-CQ is unable to cross the vacuolar membrane, that is impermeable to protic bases [31,32]. This may, thus, explain the accumulation of SQR1-CQ in the cytosol of *P. falciparum* instead.

### 2.3. Comparison of SQR1-CQ with Commercial CQ-BODIPY

**Figure 3:**
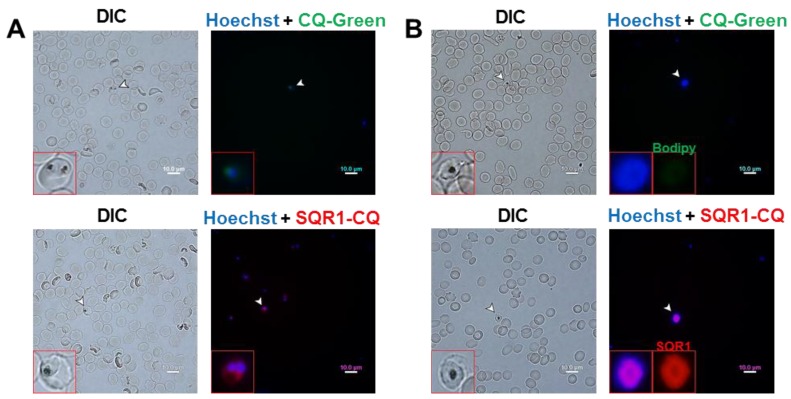
Comparison between CQ-Green and SQR1-CQ. Live 3D7 (**A**) mature trophozoites and (**B**) schizonts were co-labelled with Hoechst 33342 and either 2 µM CQ-Green or 1 µM SQR1-CQ, before analyzing via confocal microscopy on identical exposure and image capture settings. SQR1-CQ-labelled of 3D7 trophozoites and schizonts exhibited higher fluorescence than that of CQ-Green. Insets of trophozoites and schizonts indicated by arrowheads magnified at further 5×. Bar = 10 µm.

In addition, we also compared labelling of parasites with SQR1-CQ against the CQ-BODIPY conjugate studied by Loh et al. [1], which we termed CQ-Green in this study. Squaraine dyes have also been reported to be significantly brighter than BODIPY counterparts, such as in the application of fluorescence labelling of cell membranes [33], hence displaying the potential as an alternative bioimaging candidate. In this observation as demonstrated in Figure 3, *P. falciparum* cells were remarkably brighter when labelled with SQR1-CQ (1 µM) as compared to CQ-Green (2 µM), even at half the labelling concentration under 1 h incubation time. Similar labelling experiments were performed on 3D7 ring-form trophozoites to compare SQR1-CQ with CQ-Green but no visible fluorescence from the BODIPY moiety of CQ-Green was detected (Figure S2). This is in contrast with our observations of clear fluorescent labelling of immature ring-form trophozoites by SQR1-CQ, previously shown in Figure 3.

Due to the absorption of the heme chromophore in UV-vis spectral region, hemoglobin is a powerful quencher of any fluorophore exhibiting fluorescence emission below 600 nm [34,35]. Detection of malaria parasites within erythrocytes via fluorescence emission below 600 nm would be interfered by the surrounding hemoglobin or labile cytosolic heme released during parasite-mediated hemoglobin degradation [34,36,37]. Hemoglobin levels are observed to be highest in earliest erythrocytic stages of *P. falciparum*, with the least amount of hemozoin crystals detected in the ring stage and highest amount in the schizont stage [38]. Thus, the difficulty in detection of fluorescence from CQ-Green (λ_ex_: 488 nm, λ_em_: 505–525 nm) in erythrocytes could be due to hemoglobin-mediated fluorescence quenching, in addition to relatively high background of green autofluorescence from biological molecules. On the other hand, hemoglobin interference may be diminished in NIR fluorescence from SQR1-CQ (λ_ex_: 610 nm, λ_em_: 650–670 nm), owing to lower absorbance of hemoglobin in NIR spectral region. Moreover, the background fluorescence from biological molecules is minimal in NIR spectral region. Taken together, these factors confer specific advantages on NIR dyes for highly sensitive fluorescence imaging of malaria parasites.

### 2.4. IC50 Studies 

**Figure 4:**
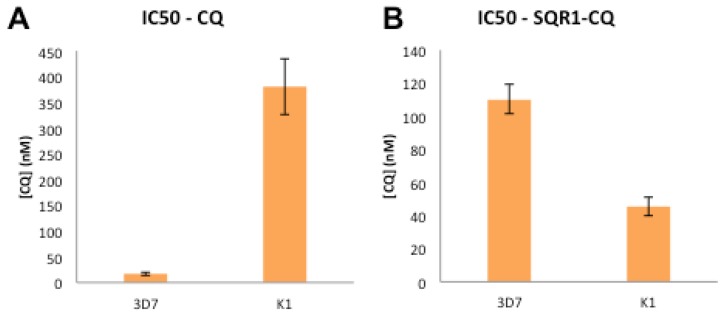
Anti-*P. falciparum* efficacy by IC50 calculation. (**A**) IC50 of CQ against resistant strain K1 (379.83 nM ± 54.62) over 20-fold higher than IC50 of CQ (16.27 nM ± 3.73) against chloroquine- sensitive strain 3D7. (**B**) SQR1-CQ IC50 against chloroquine-resistant *P. falciparum* laboratory strain K1 (45.5 nM ± 5.60) nearly 3-fold lower than SQR1-CQ IC50 against 3D7 (110.37 nM ± 9.21). Data shown are mean IC50 values from at least 3 independent experiments. Error bars indicate standard error of the mean.

The conjugation of SQR1 to CQ has provided renewed application value for CQ, in light of high resistance to CQ in malaria worldwide. In this context, we made the unexpected finding from reinvasion assays to calculate IC50 values of SQR1-CQ, that the IC50 of SQR1-CQ against resistant strain K1 is almost 10-fold lower than that of untagged CQ against K1 (Figure 4). This is in contrast to the increase in IC50 of SQR1-CQ against sensitive strain 3D7, compared to the IC50 of CQ against 3D7. While these findings go against the general trend of antimalarial activity of CQ against 3D7 and K1 [1], it is to be noted that the IC50 of SQR1-CQ against 3D7 is similar to that reported by Loh et al. for CQ-Green [1]. The lowered IC50 of SQR1-CQ against K1 might be due to a sensitizing effect mediated by the conjugation of the squaraine dye to CQ, possibly resulting in cytotoxicity against *P. falciparum* previously not observed in CQ. The K1 strain harbors a mutant *P. falciparum* CQ resistance transporter (PfCRT) that confers CQ resistance and it was recently reported that CQ resistance-conferring isoforms of PfCRT also render the parasite hypersensitive to other pharmacons [39]. Modification of CQ with SQR1 might have had such an effect in the K1 strain and further investigations should assess this hypothesis. The lower IC50 values of SQR1-CQ, compared to that of untagged CQ, against resistant strains K1 suggested renewed potential in the use of CQ as an antimalarial with added diagnostic functionality.

### 2.5. Fluorescence Staining on Unfixed Thin Blood Smears

**Figure 5:**
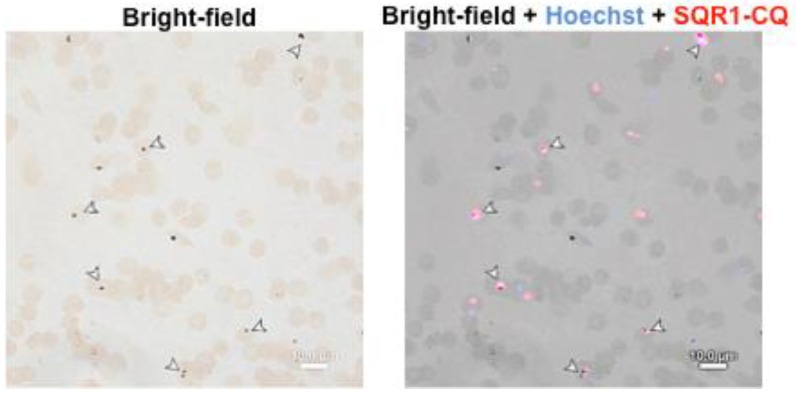
Hoechst-SQR1-CQ co-staining of unfixed thin smears of 3D7 parasites on glass slides. Thin blood smears of 3D7 parasites were prepared on clean glass slides. Prepared glass slides were co-labelled with Hoechst 33342 and 1 µM of SQR1-CQ, before analyzing via confocal microscopy. Co-localization of SQR1-CQ and Hoechst 33342 in 3D7 parasites (arrowheads). Bar = 10 µm.

Fluorescence labelling to complement the “gold standard” light microscopy methods have been recently described and assessed under field conditions [40,41], using acridine orange (AO), a nuclear stain that generally stains DNA and RNA, together with light-emitting diode (LED) fluorescence microscopy. In addition to SQR1-CQ labelling of *P. falciparum* parasites in suspension, we have also, for the first time, demonstrated directly visible SQR1-CQ and Hoechst co-labelling of *P. falciparum*-infected RBCs on unfixed thin blood smears, under a fluorescence microscope (Figure 5). This technique could be easily translated to on-site rapid diagnostic, which is deemed useful in malaria-affected developing countries with limited resources.

Besides the promising re-purpose of CQ from an antimalarial to diagnostic tool, further steps could be done to integrate SQR1-CQ into the LED fluorescence microscopy methodology, given the reported field feasibility of LED fluorescence microscopy, usability in daytime, point-of-care settings, as well as lower set-up costs and power consumption [40,41]. Moreover, the molecular structure of SQR1-CQ and the staining protocol could be enhanced further to improve the labelling concentration into the nanomolar range, previously achieved for other squaraine dye candidates [33].

## 3. Materials and Methods 

### 3.1. General Procedures

The NMR spectra were recorded on a Bruker 400 Ultrashield (400-MHz) spectrometer (Bruker, Fallanden, Switzerland), Analytical TLC was performed on commercial plates coated with silica gel (TLC MERK, silica gel 60 F254, Merk, Darmstadt, Germany). MS spectra were measured with a liquid chromatograph-mass spectrometer (LCMS 2020; Shimadzu, Kyoto, Japan).

### 3.2. Synthesis of Compounds

Compound **1**, **2**, **4**, **5** and **6** were prepared using previously reported literature methods [21,22,23,24,26,27]. 

SQR1: Squaric **3** (30.1 mg, 0.1 mmol) and indolium iodide **4a** (35.9 mg, 0.1 mmol) was refluxed for 12 h in 2.5 mL of ^*n*^BuOH/toluene (4:1 v/v) with a Dean-Stark condenser. After the solution was cooled to ambient temperature, the reaction mixture was concentrated *in vacuo* and purified via a short silica plug to obtain the butyl ester. The butyl ester was dissolved in 2 mL of 5 N HCl/THF (1:1 v/v) and refluxed for 3 h. After cooling to room temperature, the aqueous layer was extracted with CH_2_Cl_2_ (5 mL × 3), washed with brine, dried in Na_2_SO_4_ and concentrated *in vacuo*. The crude mixture was purified by preparative liquid chromatography (acetonitrile/water = 99:1) to afford a blue solid (25.0 mg, 49% yield); ^1^H NMR (400 MHz, CDCl_3_) *δ* 8.23 (d, *J* = 8.6 Hz, 2H), 7.46–7.36 (m, 2H), 7.30–7.24 (m, 2H), 6.66 (d, *J* = 8.7 Hz, 2H), 6.53 (s, 1H), 4.55 (s, 2H), 3.37 (t, *J* = 7.8 Hz, 4H), 2.91 (s, 2H), 1.83 (s, 6H), 1.61 (t, *J* = 7.8 Hz, 4H), 1.38 (q, *J* = 7.5 Hz, 4H), 0.97 (t, *J* = 7.3 Hz, 6H); ^13^C NMR (100 MHz, CDCl_3_) *δ* 185.44, 180.53, 175.26, 172.74, 151.57, 142.98, 141.10, 131.56, 128.36, 125.79, 122.55, 118.61, 111.80, 110.78, 90.13, 50.90, 50.54, 40.46, 29.70, 29.55, 26.48, 20.25, 13.90; MS (ESI) *m*/*z* (M^+^) 515.

SQR2: Compound SQR2 was prepared and purified in a similar manner as SQR1 to yield a blue solid (24.4 mg, 45% yield); ^1^H NMR (400 MHz, CDCl_3_) *δ* 8.31 (d, *J* = 8.8 Hz, 2H), 8.20–8.10 (m, 2H), 7.13 (d, *J* = 8.4 Hz, 1H), 6.70 (d, *J* = 9.0 Hz, 2H), 6.26 (s, 1H), 4.16–4.12 (m, 2H), 3.41 (t, *J* = 7.8 Hz, 4H), 1.85–1.80 (m, 6H), 1.68–1.59 (m, 4H), 1.49–1.35 (m, 8H), 1.00–0.95 (m, 9H); ^13^C NMR (100 MHz, CDCl_3_) *δ* 187.88, 178.40, 173.99, 169.80, 152.24, 146.07, 142.67, 132.11, 131.14, 126.13, 124.22, 118.94, 112.04, 109.90, 89.91, 51.02, 49.87, 44.20, 29.57, 26.83, 20.26, 13.92, 13.83; MS (ESI) *m*/*z* (M^+^) 543. 

CQ **7**: **5** (1.00 g, 4.0 mmol), triethylamine (1.12 mL, 8.0 mmol) and **6** (1.43 g, 6.0 mmol) were stirred together in a single neck flask under N_2_ atmosphere in 3 mL of dry DMF. The reaction mixture was heated to 80 °C under inert atmosphere for 12 h. After confirmation of formation of product by TLC, the residue was purified by flash chromatography to yield **7** (1.38 g, 85%). ^1^H NMR (400 MHz, CDCl_3_): *δ* 8.48 (d, *J* = 5.5 Hz, 1H), 7.95 (d, *J* = 2.2 Hz, 1H), 7.81 (d, *J* = 8.9 Hz, 1H), 7.34 (dd, *J* = 8.9, 2.1 Hz, 1H), 7.19 (s, 1NH), 6.28 (d, *J* = 5.5 Hz, 1H), 3.67 (t, *J* = 5.5 Hz, 2H), 3.41–3.38 (m, *2*H), 3.30–3.23 (m, 6H), 1.50 (s, 9H), 1.44 (s, 2H), 1.14 (t, *J* = 7.1 Hz, 3H); ^13^C NMR (100 MHz, CDCl_3_) *δ* 158.21, 150.87, 147.78, 135.36, 127.34, 125.44, 122.26, 117.01, 97.81, 80.70, 77.25, 45.48, 44.40, 43.08, 32.87, 28.43, 28.36, 13.79. 

SQR1-CQ: To a solution of **7** (44.8 mg, 0.11 mmol) in 3 mL of CH_2_Cl_2_, TFA (8.5 µL, 0.11 mmol) was added at 0 °C and allowed to stir at room temperature for 3 h. After which, the mixture was treated with HATU (76.0 mg, 0.2 mmol), 2M DIPEA (0.15 mL, 0.3 mmol) and SQR1 (51.5 mg, 0.1 mmol), before stirring at room temperature for 20 h. The mixture was diluted with CH_2_Cl_2_ (3 mL) and washed with H_2_O and brine, dried in Na_2_SO_4_ and concentrated *in vacuo*. The crude mixture was purified by preparative liquid chromatography (acetonitrile/water = 99:1) to afford a blue solid (36.2 mg, 45% yield); MS (ESI) *m*/*z* (M^+^) 803.

SQR2-CQ: Compound SQR2 was prepared and purified in a similar manner as SQR1-CQ to yield a blue solid (34.9 mg, 42%); MS (ESI) *m*/*z* (M^+^) 832.

### 3.3. Preparation of Labelling Reagents

Chloroquine diphosphate (Sigma-Aldrich, Saint Louis, MO, USA) was dissolved in PBS to a working concentration of 1 mM. LynxTag-CQGREEN (CQ-Green, BioLynx Technologies, Brockville, ON, Canada) and squaraine dyes synthesized in-house were dissolved in DMSO to a working concentration of 1 mM. All dissolved compounds were stored at −20 °C and protected from light. All dilutions of CQ, CQ-Green and SQR1-CQ were made with complete culture media.

### 3.4. Parasite Culture and Synchronization

*P. falciparum* laboratory strains 3D7 (MRA-102), K1 (MRA-159) and 7G8 (MRA-154) were obtained from MR4, ATCC Manassas Virginia (Manassas, VA, USA). All laboratory strains were cultured in complete culture media. Complete culture media consisted of RPMI 1640 (Life Technologies, Carlsbad, CA, USA) supplemented with 0.5% (w/v) Albumax I (Life Technologies, Carlsbad, CA, USA), 0.005% (w/v) hypoxanthine (Gibco, Carlsbad, CA, USA), 0.03% (w/v) L-glutamate (Sigma-Aldrich, Saint Louis, MO, USA), 0.25% (w/v) gentamycin (Gibco, Carlsbad, CA, USA), with human erythrocytes at 2.5% hematocrit. Cultures housed in tissue culture flasks were gassed with a filtered gas mixture of 3% CO_2_, 4% O_2_ and 93% N_2_ and incubated at 37 °C. Before synchronization of parasite cultures, they were centrifuged to pellet all erythrocytes and to remove culture media supernatant. To initiate synchronization, cell pellets were re-suspended in 5% (w/v) D-sorbitol (Sigma-Aldrich, Saint Louis, MO, USA) and incubated at 37 °C for 10 min. Re-suspended cells were washed twice in complete culture media, re-suspended in complete culture media and returned to culture conditions. Thin Giemsa smears were prepared and use before every experiment to determine parasitemia and parasite stage.

### 3.5. Assessment of Parasite Fluorescence Labelling via Confocal Imaging

The labelling protocol of parasites with conjugate dyes was adapted from Loh et al. [1]. 200 µL *P. falciparum* cultures at 3% parasitemia and 1.25% hematocrit were incubated with either CQ-Green or SQR1-CQ for 1–2 h at 1–2 µM and incubated at culture conditions and protected from light. After incubation, cultures were washed twice with 1 × PBS via centrifugation and stained with Hoechst 33342, as in the IC50 assay. Wet mounts of stained parasites were visualized under ×60 magnification with the Fluoview FV1000 confocal microscope (Olympus, Olympus, Tokyo, Japan). Hoechst and CQ-Green were excited at 405 nm and 488 nm with emissions captured at 430–470 nm and 505–525 nm respectively. SQR1-CQ was excited at 610 nm and emissions captured at 650–670 nm. Captured images were analyzed using FV-10 Fluoview-ASW (Olympus, Tokyo, Japan) and imageJ software (National Institutes of Health, Bethesda, MD, USA) by calculation of corrected total cell fluorescence (CTCF) from imaged fluorescent parasites, according to the following equation:

CTCF = Integrated Density − (Area of selected cell × Mean fluorescence of background readings)
(1)

### 3.6. Fluorescence Labelling of Unfixed Thin Smear Preparations

To perform dual-labelling of SQR1-CQ and Hoechst 33342, 3D7 *P. falciparum* cultures at 3% parasitemia and 1.25% hematocrit were used to prepare thin smears in clean glass slides. Thin smears were briefly air-dried before being completely immersed in solution of 1 µM SQR1-CQ for 1 h at room temperature in the dark. After incubation, SQR1-CQ labelled thin smears were washed twice with PBS by successive immersions. Washed thin smears were then immersed in 1 mg/mL Hoechst 33342 (Life Technologies, Carlsbad, CA, USA) for 20 min at room temperature in the dark. Dual labelled cells were washed twice in PBS and briefly air-dried before fluorescence image capture via confocal microscopy using similar excitation and emission parameters for SQR1-CQ and Hoechst 33342 as previously described.

### 3.7. P. falciparum Erythrocyte Invasion Half-Maximal Inhibitory Concentration (IC50)

This assay protocol was adapted from Loh et al. [1]. Briefly, *P. falciparum* cultures were synchronized at ring-stage and adjusted by dilution with complete culture media to be at 1–2% parasitemia and 1.25% hematocrit. These synchronized cultures were incubated with either CQ or SQR1-CQ at a range of concentrations, prepared via serial dilutions using complete culture media, for 48 h in 96-well flat-bottomed plates (Greiner Bio-One, Kremsminster, Austria) at culture conditions. After incubation, cultures were washed twice with 1 × PBS via centrifugation to pellet cells and to remove culture media supernatant. Washed cells were stained with 1 mg/mL Hoechst 33342 (Life Technologies, Carlsbad, CA, USA) for 20 min at room temperature in the dark. After staining, cells were washed twice and re-suspended in PBS. Parasitemia was then assessed as percentage of parasite-invaded erythrocytes after incubation, using Hoechst 33342 excitation and emission at 405 nm and 461 nm respectively with a Dako CyAn ADP flow cytometer (Beckman Coulter, Brea, CA, USA). IC50s were determined by plotting % relative parasitemia against compound concentration in Prism 5 (GraphPad, San Diego, CA, USA) software using a variable slope logistic curve.

### 3.8. Statistical Analyses

All statistical analyses were performed with GraphPad Prism 7. Fluorescence intensity comparisons between SQR1-CQ and SQR2-CQ were assessed using 2-tailed Student’s *t*-test after F-test to compare variances of samples under the two conditions.

## 4. Conclusions

In the present study, we have designed and synthesized a squaraine NIR dye, which is functionalized with chloroquine. Our assessment of the squaraine dye-CQ conjugate, SQR1-CQ, demonstrates that CQ, an antimalarial with greatly diminished therapeutic efficacy against *P. falciparum*, may be modified to be a useful tool in malaria diagnostics, one that may even have renewed antimalarial efficacy against CQ-resistant strains. SQR1-CQ is versatile and could stain very well at ring, trophozoite and schizont stages, as demonstrated in 3D7, 7G8 and K1 *P. falciparum* strains. The bright fluorescent signal from the squaraine dye, even when used at a very low dosage of 1 µM, provides a robust reporter that holds potential in expediting classical malaria diagnostic workflows by microscopy. Taken together, SQR1-CQ and future optimized variants of the conjugate may be useful new tools in detection of the *Plasmodium* parasite. The encouraging IC50 result against resistant strains K1 suggested possible growth towards future antimalarial drug development.

## Supplementary Materials

The following are available online, MS and NMR spectra, Figure S1: Comparison between SQR1-CQ and SQR2-CQ, Figure S2: CQ-Green labelling of 3D7 ring trophozoites.
