# Supplementary material for: Near Infrared Fluorophore-Tagged Chloroquine in Plasmodium falciparum Diagnostic Imaging
Source: Molecules. 2018 Oct 14;23(10):2635. doi: 10.3390/molecules23102635 (PMC6222297; doi:10.3390/molecules23102635)
Supplement: Supplementary file 1 [file molecules-23-02635-s001.pdf]

Near Infrared Fluorophore-tagged Chloroquine in *Plasmodium falciparum* Diagnostic Imaging

Li Yan Chan [1], Joshua Ding Wei Teo [1], Kevin Shyong-Wei Tan [2], Keitaro Sou [3], Wei Lek Kwan [4], and Chi-Lik Ken Lee [1,*]

1 Department of Technology, Innovation and Enterprise (TIE), Singapore Polytechnic, 500 Dover Road, Singapore 139651, Singapore; CHAN_Li_Yan@sp.edu.sg (L.Y. Chan); Joshua_TEO@sp.edu.sg (J.D.W. Teo)
2 Laboratory of Molecular and Cellular Parasitology, Department of Microbiology and Immunology, National University of Singapore, 5 Science Drive 2 Block MD4, Level 3, Singapore 117545, Singapore; mictank@nus.edu.sg
3 Research Institute for Science and Engineering, Waseda University, 3-4-1 Ohkubo, Shinjuku-ku, Tokyo 169-8555, Japan; soukei@aoni.waseda.jp
4 Engineering Product Development, Singapore University of Technology and Design, 8 Somapah Road, Singapore 487372, Singapore; kwanwl@sutd.edu.sg
* Correspondence: Ken_LEE@sp.edu.sg; Tel.: +65-6870-4891

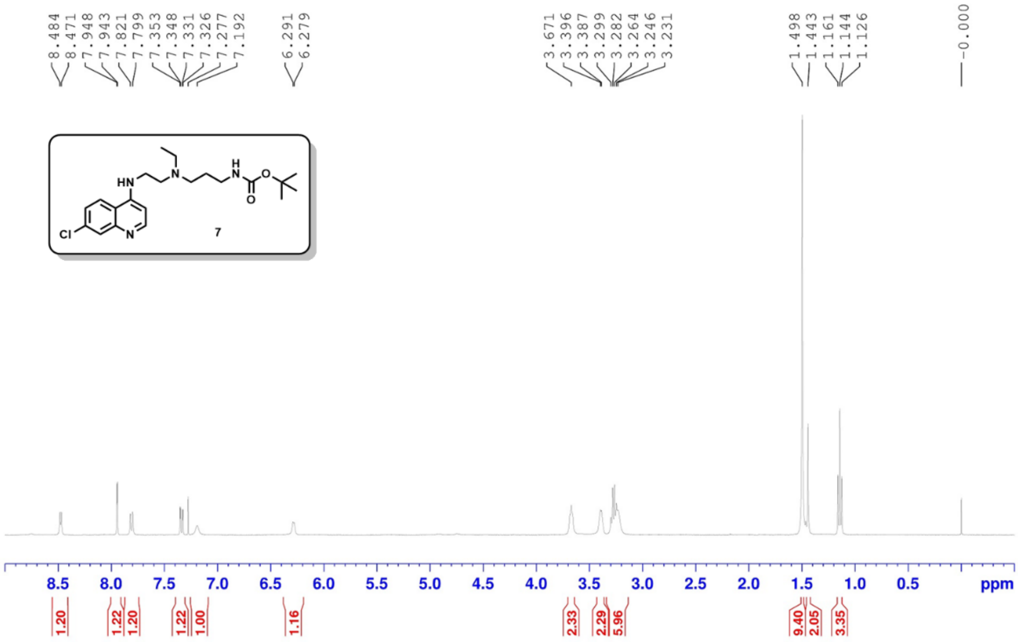
8.484
8.471
7.948
7.943
7.821
7.799
7.353
7.348
7.331
7.326
7.277
7.192
6.291
6.279
3.671
3.396
3.387
3.299
3.282
3.264
3.246
3.231
1.498
1.443
1.161
1.144
1.126
-0.000
HN
N
H
N
O
O
Cl
N
7
8.5 8.0 7.5 7.0 6.5 6.0 5.5 5.0 4.5 4.0 3.5 3.0 2.5 2.0 1.5 1.0 0.5 ppm
1.20
1.22
1.20
1.22
1.00
1.16
2.33
2.29
5.96
9.40
2.05
3.35

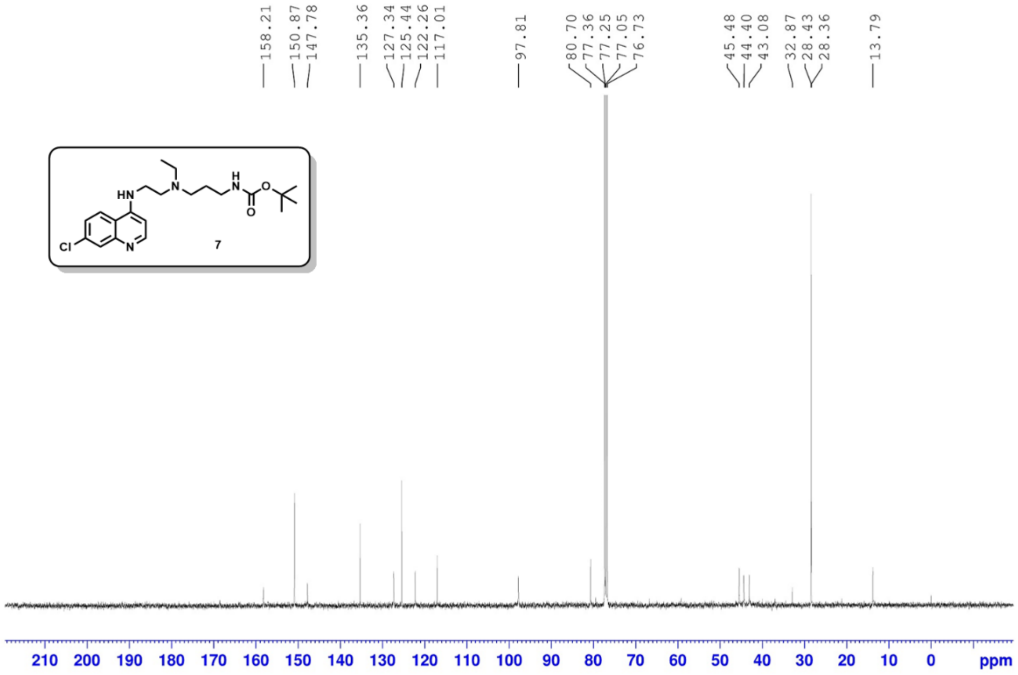
158.21
150.87
147.78
135.36
127.34
125.44
122.26
117.01
97.81
80.70
77.36
77.25
77.05
76.73
45.48
44.40
43.08
32.87
28.43
28.36
13.79
HN
N
H
N
O
O
Cl
N
7
210 200 190 180 170 160 150 140 130 120 110 100 90 80 70 60 50 40 30 20 10 0 ppm

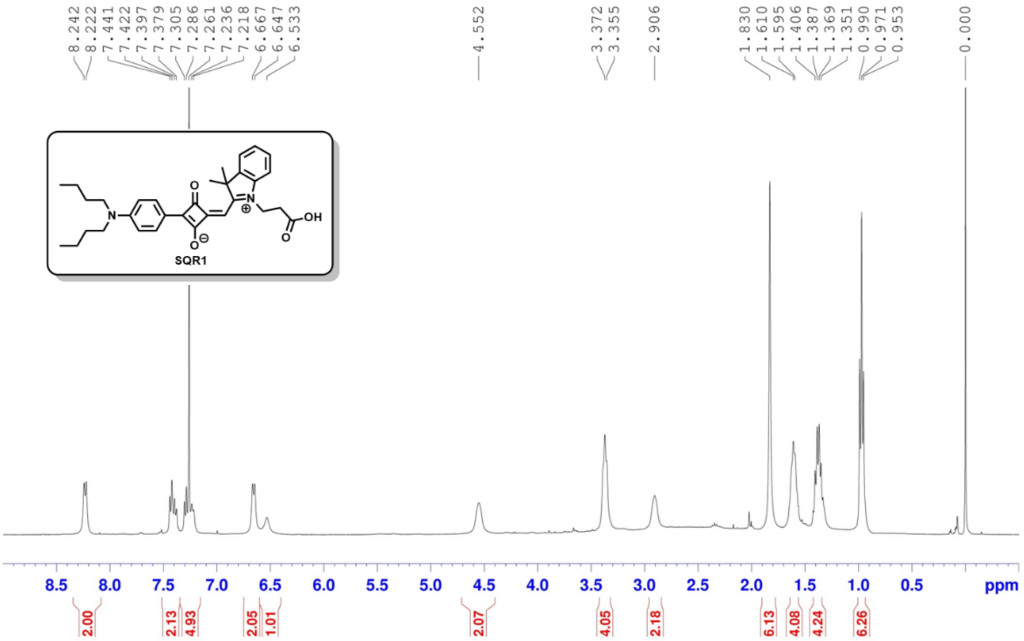
8.242
8.222
7.441
7.422
7.397
7.379
7.305
7.286
7.261
7.236
7.218
6.667
6.647
6.533
4.552
3.372
3.355
2.906
1.830
1.610
1.595
1.406
1.387
1.369
1.351
0.990
0.971
0.953
0.000
SQR1
8.5 8.0 7.5 7.0 6.5 6.0 5.5 5.0 4.5 4.0 3.5 3.0 2.5 2.0 1.5 1.0 0.5 ppm
2.00
2.13
4.93
2.05
1.01
2.07
4.05
2.18
6.13
4.08
4.24
6.26

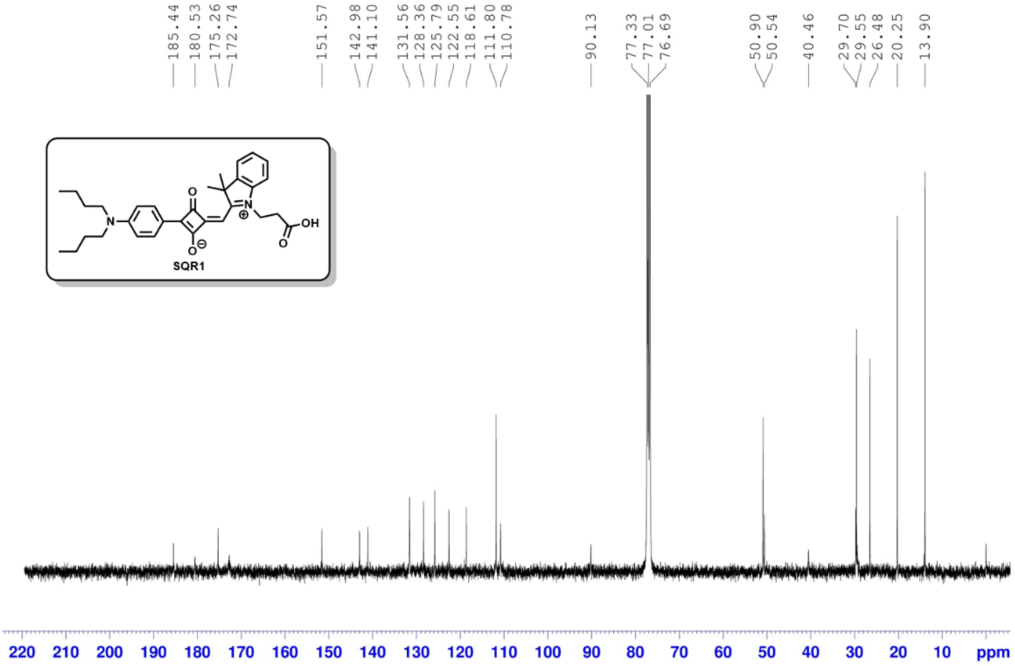
185.44
180.53
175.26
172.74
151.57
142.98
141.10
131.56
128.36
125.79
122.55
118.61
111.80
110.78
90.13
77.33
77.01
76.69
50.90
50.54
40.46
29.70
29.55
26.48
20.25
13.90
SQR1
220 210 200 190 180 170 160 150 140 130 120 110 100 90 80 70 60 50 40 30 20 10 ppm

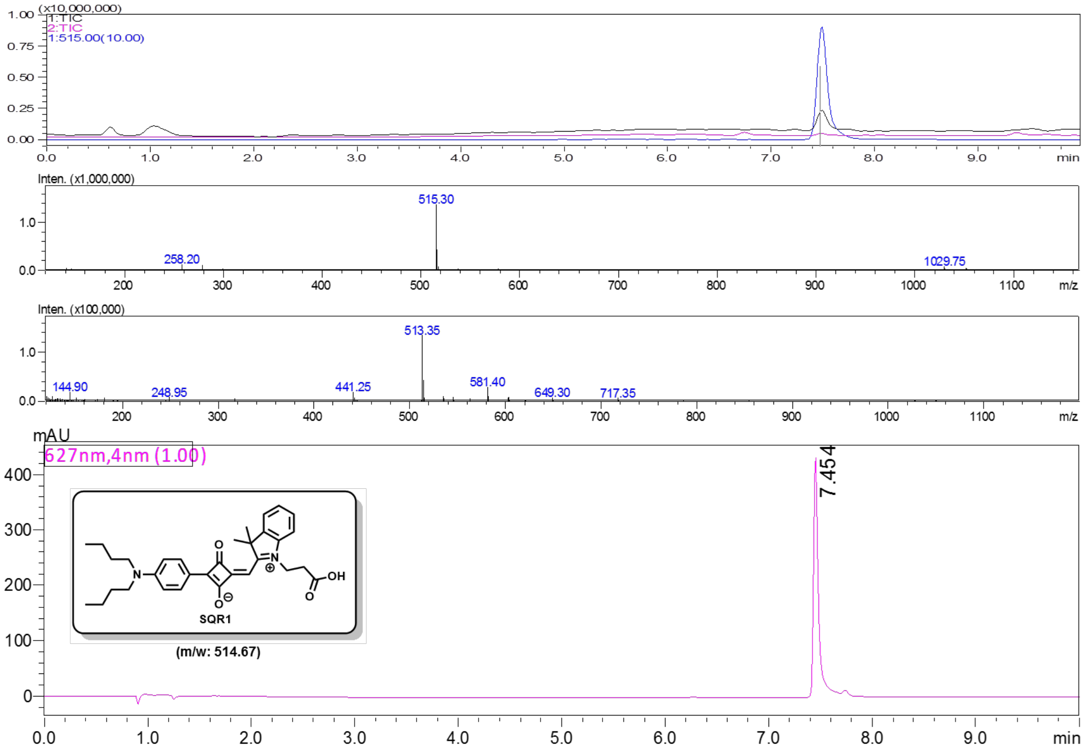

(x10,000,000)
1:TIC
2:TIC
1:515.00(10.00)
min
Inten. (x1,000,000)
515.30
258.20
1029.75
m/z
Inten. (x100,000)
513.35
144.90
248.95
441.25
581.40
649.30
717.35
m/z
mAU
627nm,4nm (1.00)
7.454
SQR1
(m/w: 514.67)
min

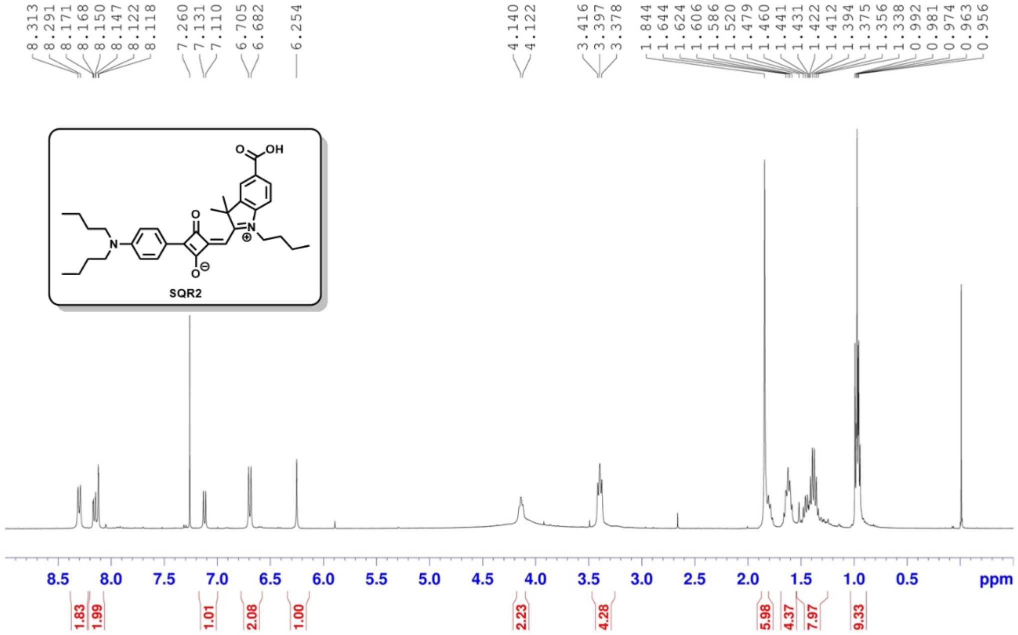

8.313
8.291
8.171
8.168
8.150
8.147
8.122
8.118
7.260
7.131
7.110
6.705
6.682
6.254
4.140
4.122
3.416
3.397
3.378
1.844
1.644
1.624
1.606
1.586
1.520
1.479
1.460
1.441
1.431
1.422
1.412
1.394
1.375
1.356
1.338
0.992
0.981
0.974
0.963
0.956
SQR2
ppm
1.83
1.99
1.01
2.08
1.00
2.23
4.28
5.98
4.37
7.97
9.33

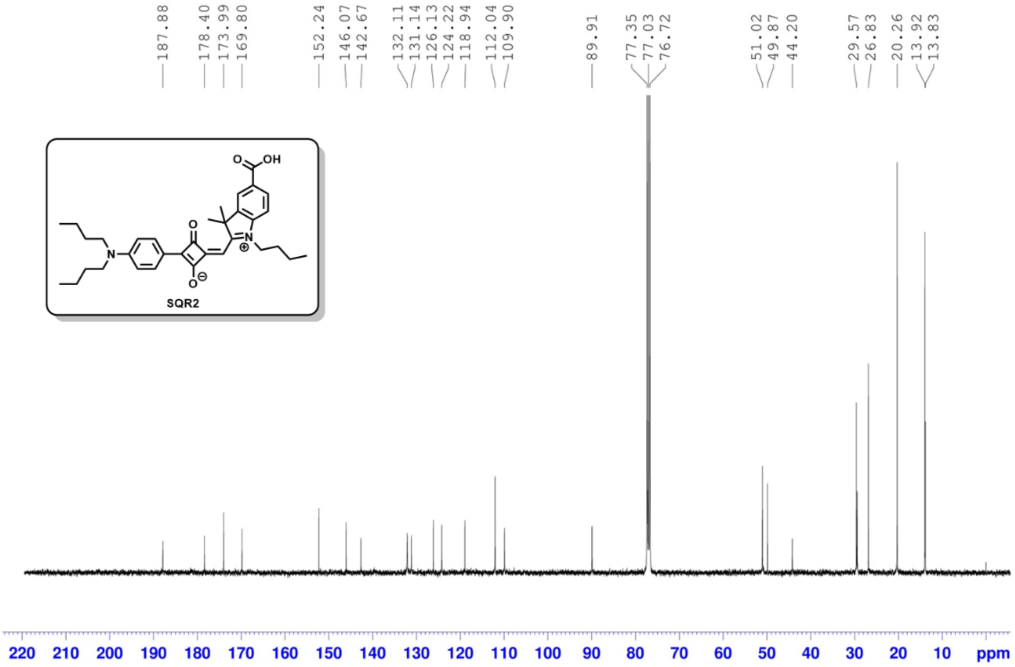
187.88
178.40
173.99
169.80
152.24
146.07
142.67
132.11
131.14
126.13
124.22
118.94
112.04
109.90
89.91
77.35
77.03
76.72
51.02
49.87
44.20
29.57
26.83
20.26
13.92
13.83
SQR2
220 210 200 190 180 170 160 150 140 130 120 110 100 90 80 70 60 50 40 30 20 10 ppm

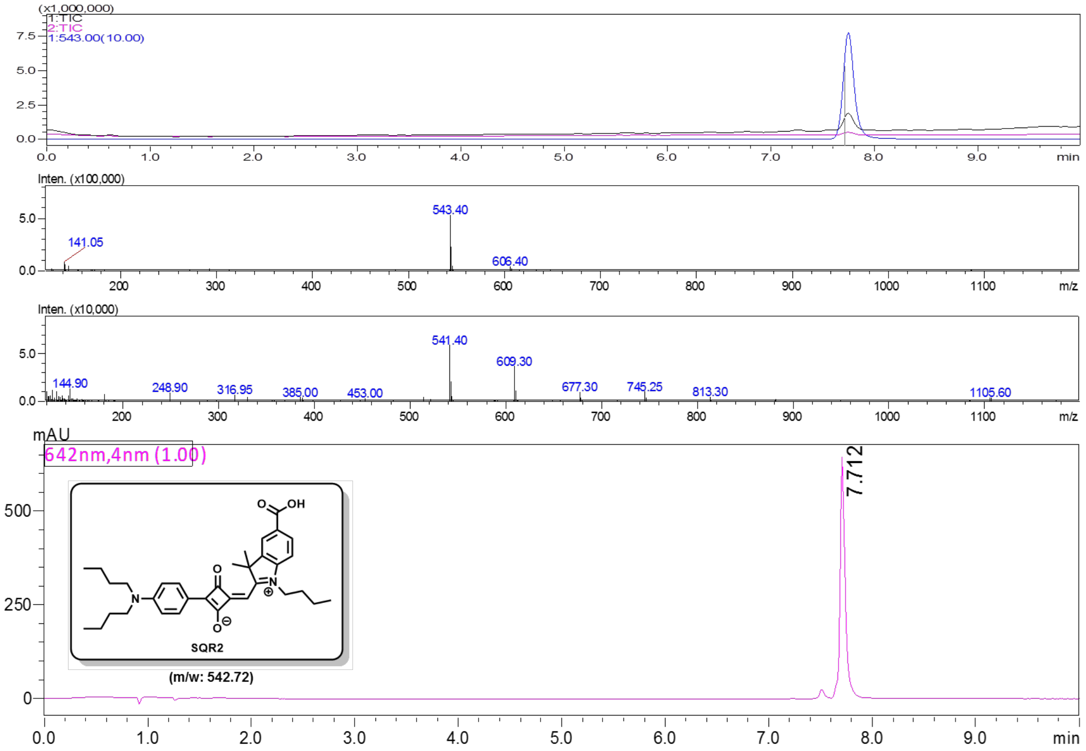
(x1,000,000)
1:TIC
2:TIC
1:543.00(10.00)
min
Inten. (x100,000)
543.40
141.05
606.40
m/z
Inten. (x10,000)
541.40
609.30
144.90
248.90
316.95
385.00
453.00
677.30
745.25
813.30
1105.60
m/z
mAU
642nm,4nm (1.00)
7.712
SQR2
(m/w: 542.72)
min

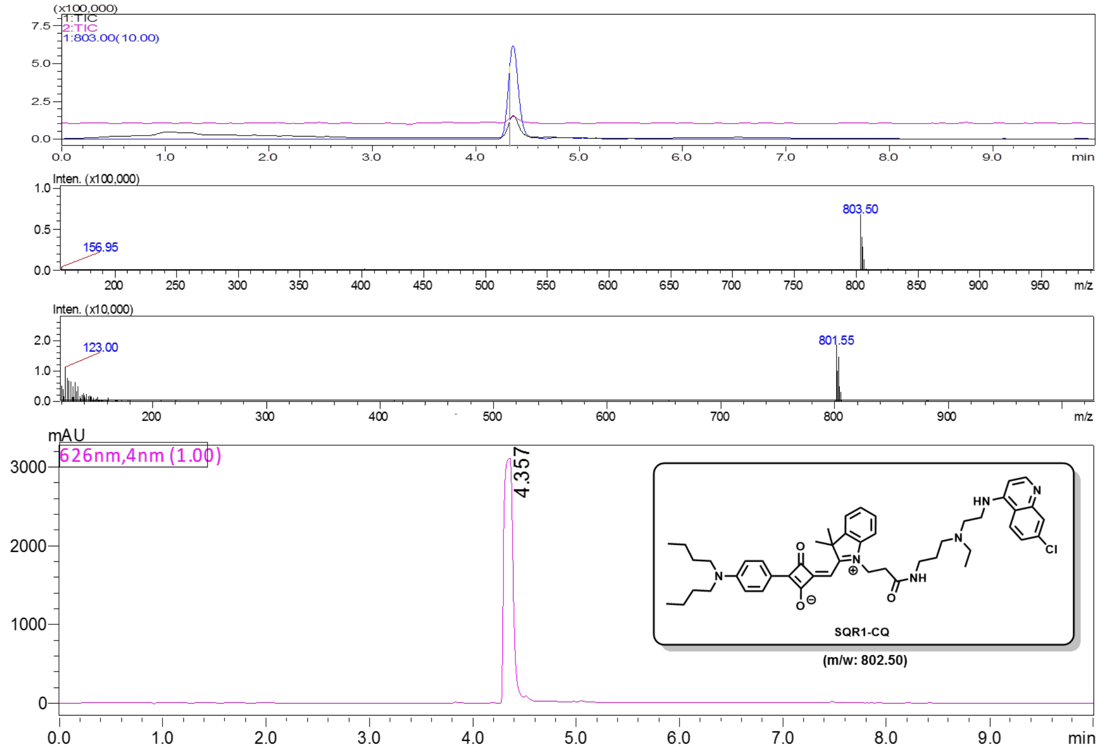
(x100,000)
1:TIC
2:TIC
1:803.00(10.00)
min
Inten. (x100,000)
803.50
156.95
m/z
Inten. (x10,000)
123.00
801.55
m/z
mAU
626nm,4nm (1.00)
4.357
min
SQR1-CQ
(m/w: 802.50)

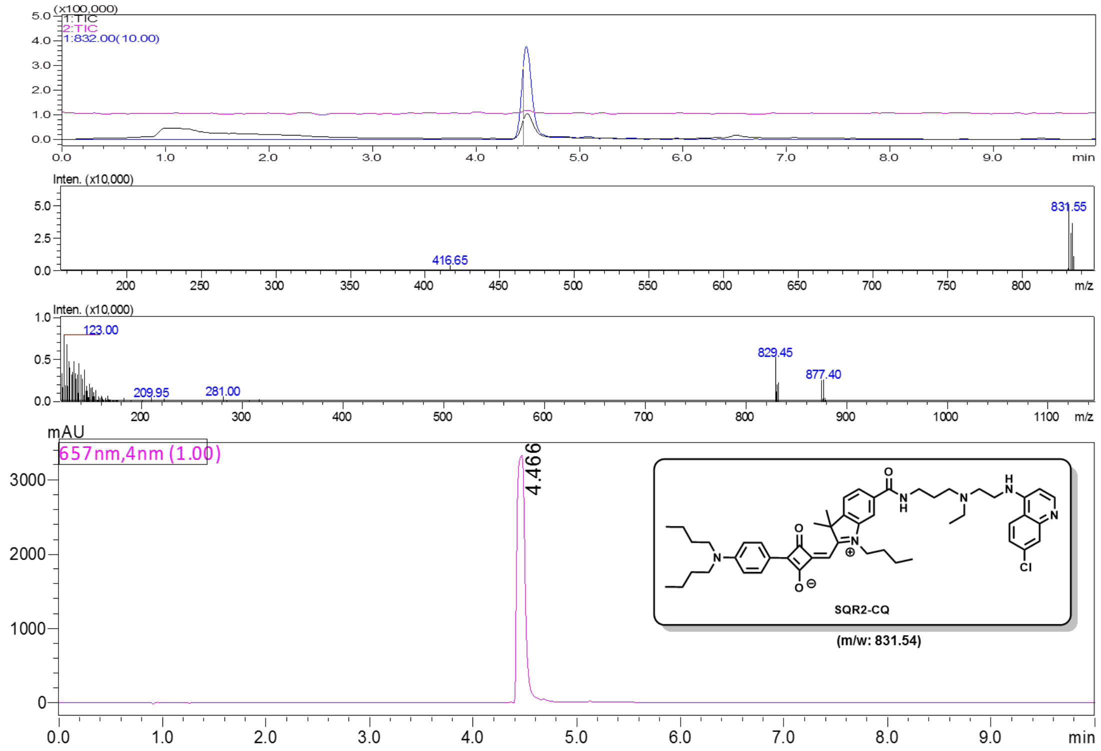
(x100,000)
1:TIC
2:TIC
1:832.00(10.00)
min
Inten. (x10,000)
831.55
416.65
m/z
Inten. (x10,000)
123.00
209.95
281.00
829.45
877.40
m/z
mAU
657nm,4nm (1.00)
4.466
min
SQR2-CQ
(m/w: 831.54)

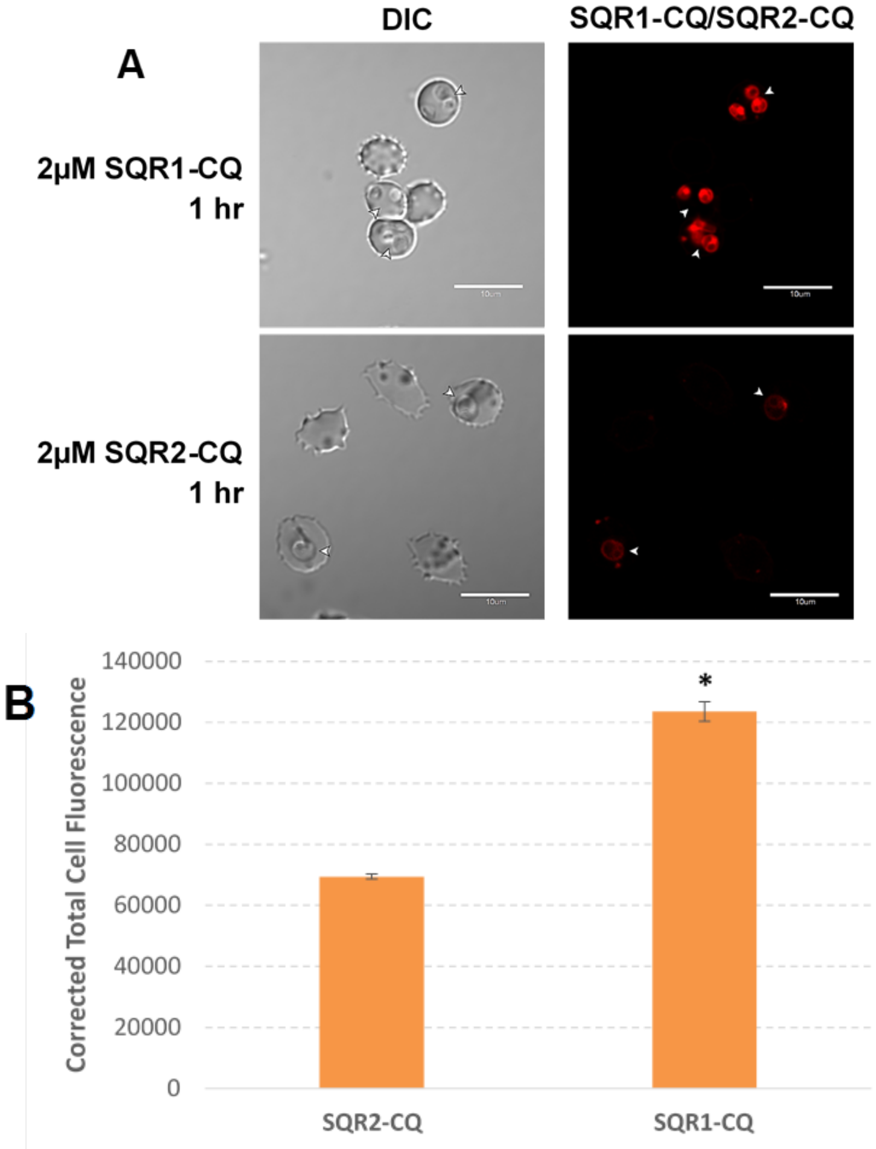

Figure S1. Comparison between **SQR1-CQ** and **SQR2-CQ**. (**A**) P. falciparum cultures were synchronised and labelled with either **SQR1-CQ** or **SQR2-CQ** and analysed via confocal microscopy. Representative confocal images of P. falciparum trophozoites labelled with either **SQR1-CQ** or **SQR2-CQ**, obtained at identical exposure and gain settings. We observe **SQR1-CQ** consistently exhibits higher fluorescence than **SQR2-CQ** (arrowheads). Both **SQR1-CQ** and **SQR2-CQ** were observed to sequester within cytoplasmic space of the parasites and not in RBCs. Surrounding unparasitized RBCs were not observed to retain **SQR1-CQ** or **SQR2-CQ**. Bar = 10 μm. (**B**) Analysis of corrected total cell fluorescence (CTCF) of trophozoites labelled with either **SQR1-CQ** or **SQR21-CQ**. Bar chart of mean CTCF from at least 3 ROI with error bars denoting standard deviation. * denotes statistical significance (p-value <0.01) compared to mean CTCF of **SQR2-CQ**.

After successful linkage of candidate squaraine compounds to CQ, **SQR1-CQ** and **SQR2-CQ** were assessed in their efficacy in fluorescence labelling of P. falciparum parasites. Both **SQR1-CQ** and **SQR2-CQ** were capable of specifically labelling only parasite cells and not the housing RBCs (Figure S1). We also did not detect **SQR1-CQ** and **SQR2-CQ** in unparasitized RBCs. Between **SQR1-CQ** and **SQR2-CQ**, we consistently observed that SQR1-CQ exhibited a stronger fluorescence signal from parasite cells than **SQR2-CQ**. We focused on further assessment of only **SQR1-CQ** for the remainder of the study.

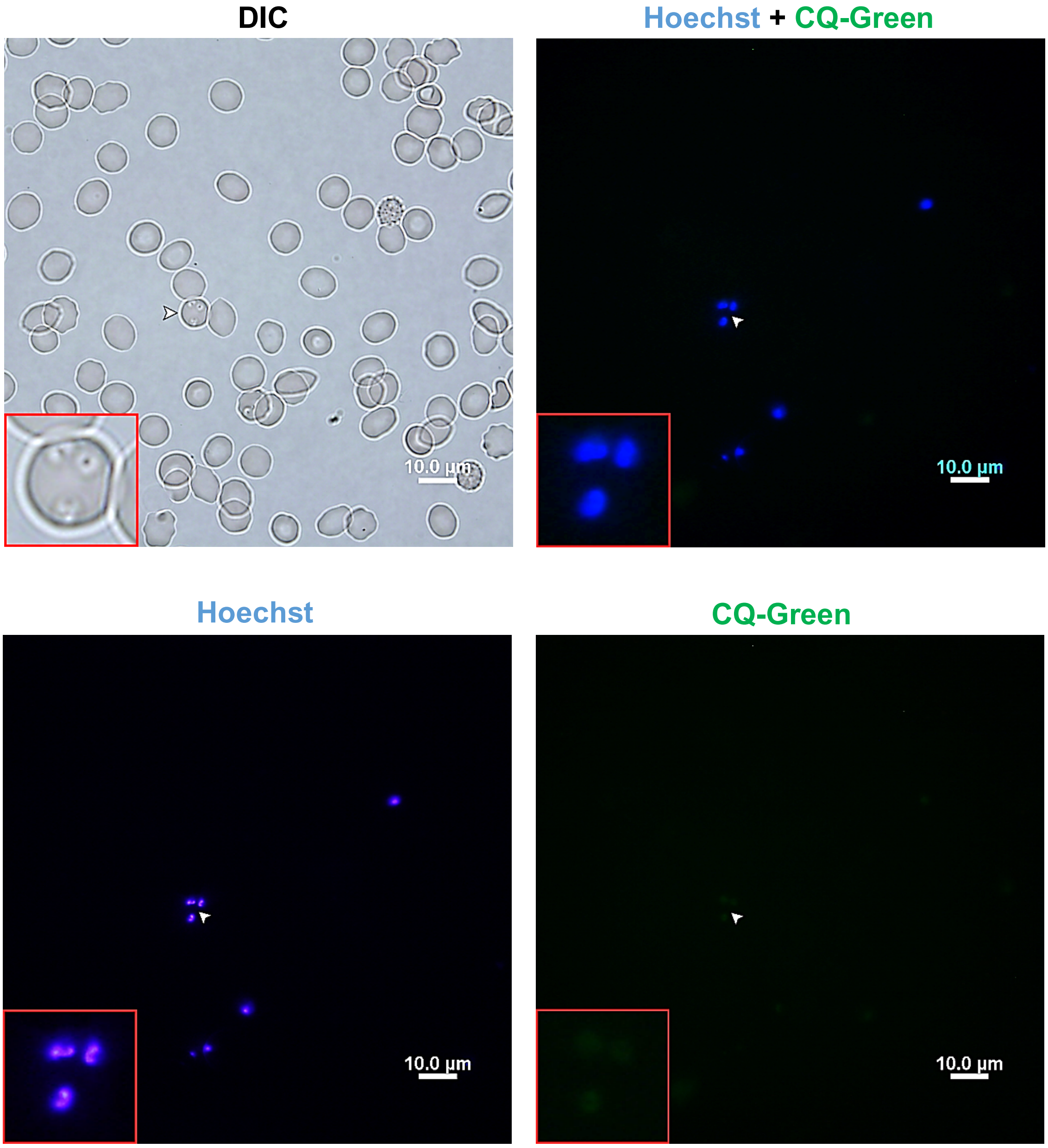

Figure S2. CQ-Green labelling of 3D7 ring trophozoites. Live 3D7 ring-form trophozoites were co-labelled with Hoechst 33342 and 2µM CQ-Green and analyzed via confocal microscopy. 3D7 rings were unable to be labelled vibrantly by CQ-Green, despite an extended incubation duration of 2 hours. Faint fluorescence signature of CQ-Green was not distinctly discernible in Hoechst-CQ-Green composite images. Insets of ring trophozoites indicated by arrowheads were magnified at further 5×. Bar = 10 µm.
